# Effects of anti-TNF-α in experimental diversion colitis^1^

**DOI:** 10.1590/s0102-865020190100000004

**Published:** 2019-12-13

**Authors:** Ronaldo Parisi Buanaim, José Aires Pereira, Fabio Guilherme Campos, Paulo Gustavo Kotze, Eduardo Felipe Kim Goto, Roberta Laís Silva Mendonça, Danilo Toshio Kanno, Carlos Augusto Real Martinez

**Affiliations:** I Fellow Master degree, Postgraduate Program in Health Science, Universidade São Francisco (USF). Assistant Professor, Faculty of Medicine, USF, Bragança Paulista-SP, Brazil. Technical procedures, acquisition and interpretation of data, manuscript preparation; USF, Faculty of Medicine, Bragança Paulista, SP, Brazil; II PhD, Assistant Professor, Division of Pathology, Faculty of Medicine, USF, Bragança Paulista-SP, Brazil. Histopathological examinations, acquisition and interpretation of data; III PhD, Associate Professor, Department of Gastroenterology, Faculty of Medicine, Universidade de São Paulo (USP), Brazil. Analysis and interpretation of data, critical revision; IV PhD, Assistant Professor, Colorectal Surgery Unit, Cajuru University Hospital, Universidade Católica do Paraná (PUCPR), Curitiba-PR, Brazil. Analysis and interpretation of data, English language revision, critical revision; V Graduate student, Faculty of Medicine, USF, Bragança Paulista-SP, Brazil. Technical procedures, acquisition of data; VI Fellow Master degree, Postgraduate Program in Health of Sciences, USF, Bragança Paulista-SP, Brazil. Technical procedures, Histopathological examinations; VII Fellow PhD degree, Postgraduate Program in Health Science, USF. Assistant Professor, Division of Surgery, Faculty of Medicine, USF, Bragança Paulista-SP, Brazil. Technical procedures, acquisition of data, manuscript preparation; USF, Faculty of Medicine, Division of Surgery, Bragança Paulista, SP, Brazil; VIII PhD, Associate Professor, Postgraduate Program in Health Sciences, USF, Bragança Paulista-SP, and Department of Surgery, Universidade Estadual de Campinas (UNICAMP), Campinas-SP, Brazil. Conception and design of the study, statistics analysis, manuscript preparation and writing, critical revision; Universidade Estadual de Campinas, Department of Surgery, Campinas, SP, Brazil

**Keywords:** Colitis, Colostomy, Fatty Acids, Tumor Necrosis Factor-Alpha, Infliximab, Rats

## Abstract

**Purpose:**

To evaluate the effects of infliximab on the inflammation of the colonic mucosa devoid from fecal stream.

**Methods:**

Twenty-four rats were submitted to a Hartmann's procedure. They remained for 12 weeks with the fecal derivation to development of diversion colitis on excluded colorectal stump. After this period, they were divided into 3 groups: one group received intervention with saline (2.0 mL / week), other group infliximab at doses of 5 mg/kg/week and the other 10 mg/kg/week for five consecutively weeks. Concluded the intervention period, the animals were euthanized to remove colon segments with and without fecal stream. Colitis was diagnosed by histological analysis and the degree of inflammation by validated score. The neutrophilic infiltrate was evaluated by tissue expression of myeloperoxidase identified by immunohistochemical. The tissue content of myeloperoxidase was measured by computer-assisted image analysis.

**Results:**

The inflammatory score was high in colonic segments without fecal stream. The intervention with infliximab reduced the inflammatory score in excluded colonic segments. The content of myeloperoxidase was reduced in colonic segments of animals treated with infliximab mainly in high concentrations.

**Conclusion:**

Intervention with infliximab reduced the inflammation and the neutrophil infiltrate in colonic segments devoid of the fecal stream.

## Introduction

Diversion colitis (DC) is a benign condition characterized by the appearance of chronic inflammation in the mucosa of the colon or rectum devoid of the fecal stream^1^ ^,^ ^2^. The etiopathogenic basis for the development of DC is not yet fully understood^3^ ^,^ ^4^. Most of the authors believe that the disease is a nutritional deficiency syndrome caused by deficiency of the regular supply of short-chain fatty acids (SCFAs), the main energy substrate for the metabolism of the colonic epithelial cells^5^ ^,^ ^6^. The lack of the regular supply of SCFAs to the cells of the colonic epithelium causes modifications in energy metabolism increasing the production of reactive oxygen species (ROS)^7^. ROS are toxic to cells and their overproduction causes breakage of the various lines of defense that make up the mucosal barrier, allowing bacteria of the colon lumen to migrate to the sterile submucosa^7^ ^–^ ^9^. In an attempt to combat this bacterial infiltration, neutrophils migrate to the intestinal vessels, produce large amounts of pro-inflammatory cytokines like IL-1β, IL-6 and tumor necrosis factor alpha (TNF-a) leading to the damage of the colonic mucosa characteristic of the disease^7^.

Majority of patients with DC are asymptomatic or develop few symptoms, but it is estimated that 10%-15% of patients develop the most severe forms of the disease^4^. Many patients need to remain with the colostomy for long periods, and some will never attain the reconstruction of colonic continuity. Therefore, it is expected that development of DC will impair the quality of life in a significant number of patients^10^ ^–^ ^12^. It is estimated that about 30% of DC symptomatic patients complain of serous, bloody or mucous discharge per anus^12^. Tenesmus, fever and abdominal pain occur in 15% of the population^13^. Less frequently, patients may experience severe rectal bleeding or sepsis necessitating an emergency colectomy or extra intestinal manifestations^13^ ^–^ ^15^.

The mainly option to the treatment of DC should be primarily directed at the reconstruction of the colonic continuity to restore the normal luminal supply of SCFAs^16^. Unfortunately, the optimal treatment for DC in patients in whom fecal stream restoration cannot be performed has not yet been found. In this situation, several clinical therapeutic strategies have been proposed. The application of enemas in diverted segment of the colon or rectum with nutritional solutions rich in SCFAs or glutamine, autologous fecal transplantation, the use of enemas with anti-inflammatory or antioxidant substances (5-ASA, n-acetylcysteine, sucralfate, curcumin, and steroids) and use of oil extract of coconut with controversial results^2^ ^,^ ^17^ ^–^ ^23^. However, the need for daily application of enemas containing these substances decreases patients’ adherence to this therapeutic strategy.

When considering the clinical and histopathological similarities between inflammatory bowel diseases (IBD) and the severe forms of DC, it can be assumed that strategies used for the treatment of IBD may be valid for DC^24^ ^–^ ^25^. Reinforcing this evidence, recently, it has been shown that severe and chronic forms of CD can be a trigger for the development of IBD^15^.

Clinical studies have shown that the use of biological therapy with anti-TNF-a represents the most effective therapeutic strategy for the treatment of patients with IBD^26^. Similarly, an experimental study showed that subcutaneous application of infliximab improved inflammation in the colonic mucosa of rats with colitis induced by 2,4,6, trinitrobenzene sulfonic acid (TNBS), an experimental model of induced-colitis^27^. It has been demonstrated that in the mucosa of colonic segments devoid of fecal stream in experimental models of DC, there is an increase in the tissue content of TNF-a^7^ ^,^ ^17^. Thus, it is possible that the use of infliximab will be effective for treatment, especially in those patients with severe forms of DC. However, to the best of our knowledge, this possibility has not yet been evaluated clinically or experimentally. Thus, the objective of the present study is to evaluate the efficacy of the use of biological therapy with infliximab in an experimental model of DC.

## Methods

The accomplishment of this study obeyed Federal Law 6,638 and the guidelines of the Brazilian College of Animal Experimentation (COBEA). The study was approved by the Ethics Committee on the Use of Animal in Research of the Universidade São Francisco (number: 0102262014).

### Animals and surgical technique

Twenty-four male Wistar rats, weighing between 250 and 300g were used. In the seven days preceding the operation, the animals were confined in individual cages receiving rodent-specific food and water ad libitum. From the day before the operation, they were fasted, except water, for 12 hours. On the day of the intervention, the rats were anesthetized with ketamine (5 mg/kg) associated with xylazine (60 mg/kg) given intraperitoneally. The abdominal cavity was accessed through a median longitudinal incision with 4cm in extension. After locating the Peyer's plaque and mobilizing the fecal contents, the colon section was made 8 cm above the proximal end of the plaque. The distal segment of the sectioned large intestine was catheterized with a 10 F polyvinyl catheter and irrigated with 40 ml of 0.9% saline, at 37°C, to promote the removal of fecal waste from the distal segment. After irrigation, the distal remaining colorectal segment was closed by running stiches and the proximal segment of the colon (with fecal stream) was externalized as a terminal stoma (Hartmann's procedure). After stoma fixation at skin, the abdominal wall was closed. After recovery, the animals were released for water intake and, after six hours, standardized ration (Nuvilab CR1™ Nuvital Nutrientes AS, Brazil).

We waited 12 weeks after the surgical procedure, to develop DC as proposed by other study^9^. After randomization, the animals were divided into 3 experimental groups with 8 animals each: A-) saline (control group), B-) Infliximab at a dose of 5 mg/kg/ week and C-) infliximab at dose of 10 mg/kg/week. The solutions of intervention were given subcutaneously in the dorsal region weekly for five consecutive weeks. No additional care was taken in relation to the stoma. On the day of euthanasia, all animals were again fasted for 12 hours except for water.

After the 5 weeks of intervention with the proposed substances, all rats were anesthetized again with the same methodology previously described for realizing the diversion of the fecal stream. The anterior medial incision was reopened and the colonic segments with and without fecal transit were removed. Colonic segments with 3 cm of extension (one of the colon provided, another of the colon devoid of transit) were sent for histological and immunohistochemically studies. The animals were submitted to euthanasia by single and lethal injection of thiopental (120mg/kg).

### Histological technique

The excised specimens destined for the histological study were fixed in 10% formaldehyde for 72 hours, subsequently dehydrated in successive increasing concentrations of alcohol, and clarified in xylene. Then the material was included in paraffin blocks and subjected to longitudinal cuts, with a thickness of 4m for mounting the slides. After assembly, the slides were subsequently stained by hematoxylin-eosin (for analysis of histological changes of specimens) and immunohistochemistry for the evaluation of tissue expression of myeloperoxidase (MPO), which shows the presence of neutrophil infiltration.

The analysis of each slide was done in a common optical microscope with a final magnification of x200. The histological parameters were analyzed by means of image processing computer-analysis by a pathologist with experience in diseases of the digestive tract and who was blinded regarding the experimental group analyzed. To stratify the inflammatory degree score, the following main histological parameters were considered: epithelial atrophy, epithelial loss, presence of crypt abscess and intensity of the inflammatory neutrophil infiltration. For the stratification of each variable, the following criteria were observed: 0 = absent; + = minimum; ++ = slight; +++; moderate and, ++++ = severe. The inflammatory degree was graded from 0 to 16 crosses, 0 = absent; 1-4 mild; 5-9 moderate and >10 severe, after summation of the scores found in the variables analyzed (epithelial atrophy, epithelial loss, crypt abscess and neutrophil infiltrate).

### Immunohistochemically analysis

The immunohistochemical technique for the investigation of tissue expression of MPO was performed using a previously described methodology^22^. Briefly, all blocks were sectioned in 5µ thick sections obtained from colonic segments with or without fecal stream of the animals treated with saline or both concentration of infliximab. Slides were diaphanized and rehydrated, and antigen retrieval was performed using the Trilogy solution (Cell Mark Inc., Rocklin, CA, USA). Endogenous peroxidases were blocked using 3% hydrogen peroxide (H_2_O_2_) in a humid chamber at room temperature for 10 min and after washing were performed with PBS. The primary polyclonal anti-MPO antibody (Dako do Brazil Ltda., Sao Paulo, Brazil) with cross-reactivity to rats was diluted in saline containing bovine serum albumin (1%) diluted 1:100. All slides were coated with 100µL of this solution and left resting at room temperature for 2h. Following exposure to primary antibody, the slides were rinsed with distilled water and PBS buffer. Then, the slides were incubated with an ABC system comprising the LSAB + kit System-HRP (Dako do Brasil Ltda., Sao Paulo, Brazil) for a 35-min period of exposure for each reagent, and then washed with PBS. The section processing occurred by using the Liquid DAB + Substrate Kit (Dako do Brasil Ltda., Sao Paulo, Brazil) in a dilution of 1 drop of chromogenous solution in 1 µL of buffer solution for a period of 5 min at room temperature. After this processing, the sections were washed and counterstained with Harris hematoxylin for 30s. Finally, the slides were dehydrated with increasing concentrations of alcohol and xylene and mounted with coverslips and resin. The positive control for the presence of MPO was done using a slide obtained from a patient with acute appendicitis, while the negative control with other slide, however without adding the anti-MPO primary antibody.

### Image processing computer-analysis

The quantification of the tissue expression of MPO was measured by image processing computer-analysis. The slides of the segments with and without intestinal transit were always performed in a place where there were at least three intact and contiguous crypts. The selected image was captured by video camera pre-coupled to optical microscopy (Eclipse DS50^®^ - Nikon Inc., Japan). The captured image was processed and analyzed by the NIS-Elements software (Nikon Inc., Japan). To measure the tissue content of MPO, we used a common optical microscope, always with a final magnification of 200 times. It was considered as a positive reaction to brownish coloration that identified the presence of MPO in the neutrophils. The image analysis program, using color histograms, determined the color intensity of each area selected for measurement (neutrophils labeled by the anti-MPO antibody), transforming the chosen color (brownish) into percentage numerical expression for each field of view selected. The final value adopted for each field measured in the segments provided and devoid of intestinal transit was represented by the average values found in the evaluation of six different fields. The tissue content of MPO was classified according to the percentage of neutrophils stained by histological field (% / field).

### Statistical analysis

The results for the degree of inflammation were described according to the median of the values obtained. As to tissue levels of MPO, the results were described according to their mean ± standard error. The comparison of results found among experimental groups was analyzed by Mann-Whitney test. It was established a level of significance of 5% (p < 0.05), and was used one asterisk (*) to identify values of p ≤ 0.05 and two asterisks (**) for values of p ≤ 0.01.

## Results

**Figure 1:**
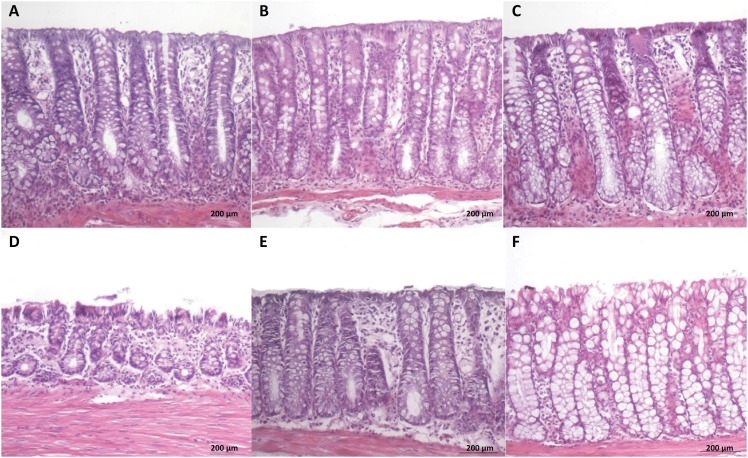
**A:** Colonic epithelium with fecal stream of animal submitted to intervention with saline. **B:** Colonic epithelium with fecal stream of animal submitted to intervention with infliximab at doses 5 mg/kg/week. **C:** Colonic epithelium with fecal stream of animal submitted to intervention with infliximab at doses 10 mg/kg/week. **D:** Colonic epithelium without fecal stream of animals submitted to intervention with saline. **E:** Colonic segment without fecal stream of animals submitted to intervention with infliximab at doses 5 mg /kg / week. **F:** Colonic segments devoid of fecal stream of animal submitted to intervention with infliximab at doses 10 mg / kg / week (HE-x200).

Figure 1 A-C shows colonic segments with fecal stream of animals submitted to intervention with saline, infliximab 5 mg /kg/ week and infliximab 10 mg /kg /week, respectively. Figure 1 D-F shows colonic segments without fecal stream of animals submitted to intervention with saline, infliximab at concentrations of 5 mg/kg/week and 10 mg/kg/week, respectively. In those animals with colonic segments provide of fecal stream, regardless of the substance used, the mucosal surface is preserved without formation of ulcers, the colonic glands are intact, and the number of goblet cells is similar among groups (Fig. 1 A-C). The colonic segments devoid of fecal stream in animals submitted to intervention with saline present's atrophy of the colonic glands, presence of epithelial erosions, edema with stromal inflammatory infiltrate and reduction in the population of goblet cells (Fig. 1D). Distinctly, the colonic segments without fecal stream of the animals submitted to intervention with infliximab, regardless of the concentration used, the length of colonic glands is maintained, with decrease of inflammatory infiltrate and increase in numbers of goblet cells (Fig. 1 E-F).

**Figure 2:**
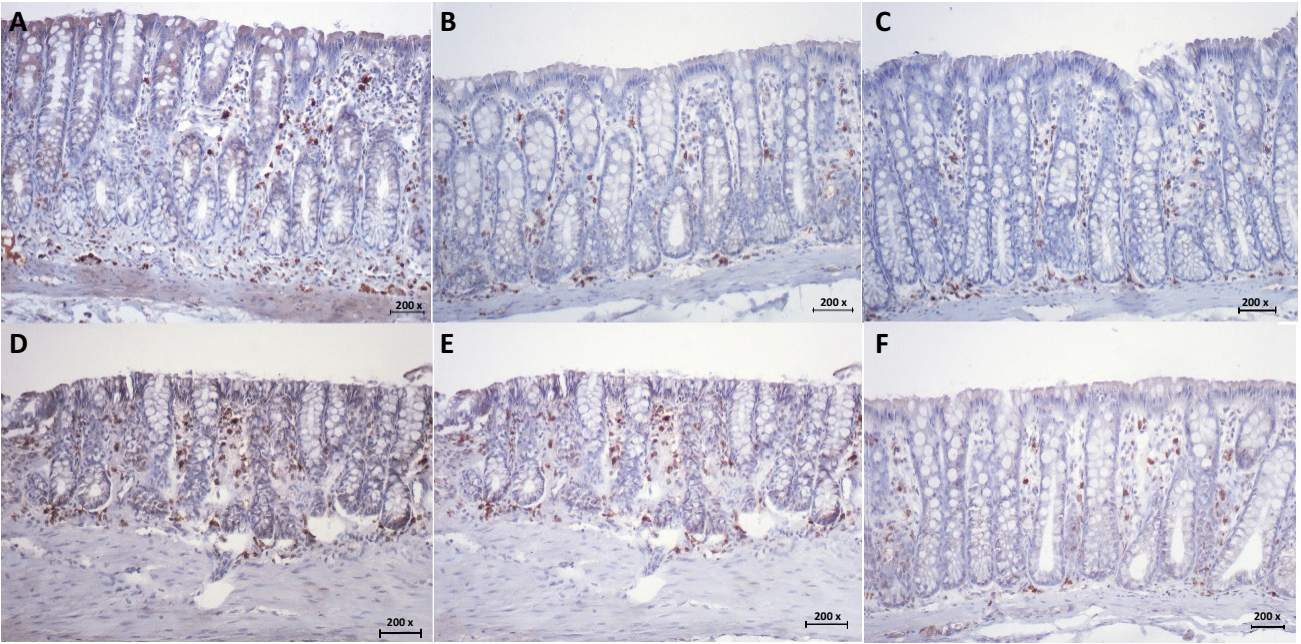
**A:** MPO tissue expression in colonic segments with fecal stream of animals submitted to intervention with saline. **B:** MPO tissue expression in colonic segments with fecal stream of animals submitted to intervention with infliximab at doses 5 mg/kg/week. **C:** MPO tissue expression in colonic segments with fecal stream of animals submitted to intervention with infliximab at doses 10 mg/kg/week. **D:** MPO tissue expression in colonic segments without fecal stream of animals submitted to intervention with saline. **E:** MPO tissue expression in colonic segments without fecal stream of animals submitted to intervention with infliximab at doses 5 mg/kg/week. **F:** MPO tissue expression in colonic segments devoid of fecal stream of animals submitted to intervention with infliximab at doses 10 mg/kg/week (IH-MPO-x200).

Figure 2 A-C shows the tissue expression of MPO in colonic segments provided of fecal stream of animals submitted to intervention with saline, infliximab 5 mg/kg/week and 10 mg/kg/week, respectively. The colonic mucosa of the animals with preserved fecal stream has small number of neutrophils (dark brown color staining), mainly located in the stroma between the colonic glands and nearly of muscularis mucosa and colonic crypts. Figure 2 D-F shows the tissue expression of MPO in colon segments without fecal stream of animals submitted to intervention with saline, infliximab at concentrations of 5 mg / kg / week and 10 mg / kg / week, respectively. In animals subjected to saline intervention (Fig. 2D), it is possible to verify an increase in the numbers of neutrophil infiltrate mainly located in the stroma between the colonic glands. In contrast, in animals undergoing intervention with infliximab, there is less neutrophil infiltration, particularly in those that received infliximab at concentration of 10 mg / kg / week (Fig. 2F).

**Figure 3:**
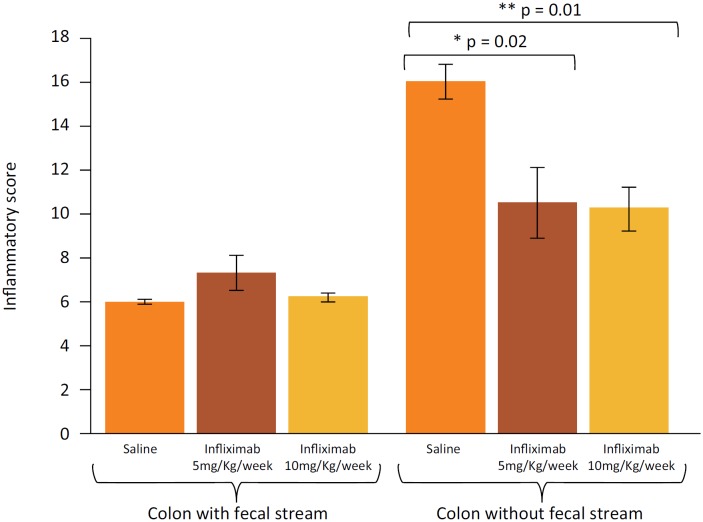
Inflammatory score in the colonic segments with and without fecal stream, in the animals treated with saline, infliximab 5 mg/kg/week and infliximab 10 mg/kg/week. * = p <0.05 (Saline > Infliximab 5 mg/kg/week); ** = p ≤ 0.01 Saline > infliximab 10 mg/kg/week). Mann-Whitney test.

Figure 3 shows the inflammatory score in colonic segments with and without fecal stream of animals submitted to intervention with saline, and infliximab at concentrations of 5 mg/kg/week and 10 mg/kg/week, respectively. In the colonic segments provided with fecal stream, no differences in inflammatory score were identified regardless of saline or infliximab intervention (5 mg/kg/week or 10 mg/kg/week). Differently, colonic segments without fecal stream of the animals submitted to intervention with saline presented a higher degree of inflammation when compared to those submitted to the infliximab intervention.

**Figure 4:**
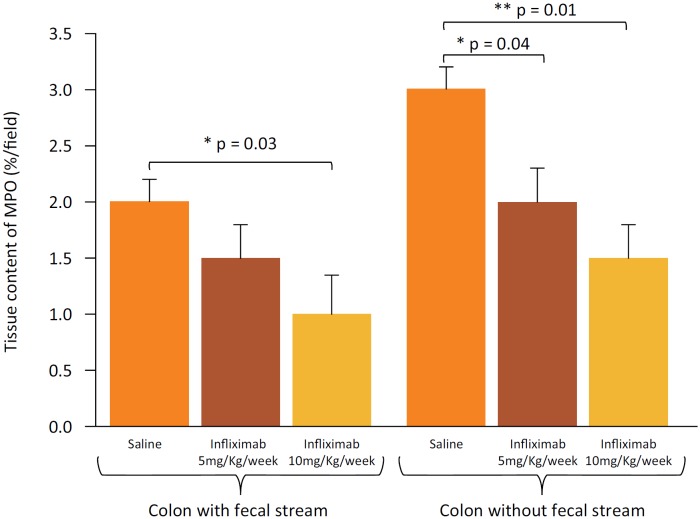
Tissue content of MPO in the colonic segments with and without fecal stream, in the animals treated with saline, infliximab 5 mg/kg/week and infliximab 10 mg/kg/week. * = p < 0.05 (Colon with fecal stream: Saline > Infliximab 10 mg/kg/week and Colon without fecal stream: Saline > infliximab 5 mg / kg / week). ** = p ≤ 0.01 (Colon without fecal stream: Saline > Infliximab 10 mg/kg/week). Mann-Whitney test.

Figure 4 shows the tissue content of MPO in colonic segments with and without fecal stream of animals submitted to intervention with saline, and infliximab at concentrations of 5 mg/kg/weeks and 10 mg/kg/week, respectively. In the colonic segments provided with fecal stream, the neutrophil infiltrate reduced only in those animals submitted to intervention with infliximab at concentration of 10 mg/kg/week. In the colonic segments without fecal stream, the neutrophil infiltrate was significantly lower in those animals submitted to intervention with infliximab regardless of the concentration used.

## Discussion

Studies using different models of chemically induced colitis have shown that there is an increase in the production of TNF-α in the inflamed colonic mucosa^24^ ^,^ ^27^ ^,^ ^28^. In order to verify if the biological therapy would be able to improve the induced colitis, some authors evaluated the effectiveness of the application of anti-TNF-α in these experimental models of colitis^25^ ^,^ ^27^ ^,^ ^28^. The results of these studies showed that the biological therapy with anti-TNF-α antibodies was effective in improving the inflammatory tissue process and reducing the tissue levels of TNF-α. In these models of chemically induced colitis, subcutaneous application of anti-TNF-α at doses of 5 mg/kg/week and 10 mg/kg/week reduced the inflammatory activity score in the colonic mucosa, TNF-α levels, and tissue levels of malondialdehyde, an important marker of tissue oxidative stress. Other authors, with the objective of verifying whether anti-TNF-α use associated with antioxidants was effective for the treatment of chemically induced colitis by dextran sulfate, also showed that the use of anti-TNF-α antibodies reduced levels of TNF-α and improved the inflammatory process of the colonic mucosa^29^.

Although the restoration of fecal transit is an effective strategy for the treatment of DC in the most severe forms, it becomes a risky procedure because a surgical anastomosis will be performed in a chronically inflamed intestinal segment. In order to correct the mucosal inflammatory process that occurs in colonic segments devoid of fecal transit that develop DC, therapeutic strategies like those employed for the treatment of IBD have been proposed. Thus, the application of enemas with 5-ASA (mesalazine), n-acetylcysteine, corticosteroids and others with natural substances with antioxidant properties have shown to be effective in the treatment of mild and moderate forms of DC^2^ ^,^ ^17^ ^–^ ^23^. These studies attribute the efficacy of these substances to their antioxidant and anti-inflammatory properties. However, these drugs have therapeutic effects on mild and moderate forms of DC and need to be applied by enemas.

Although anti-TNF-α therapy represents the most effective strategy for the treatment of patients with IBD, few experimental studies have evaluated the substance use in experimental models of chemically induced colitis^27^ ^–^ ^30^. The results of these studies showed that intraperitoneal or subcutaneous use of anti-TNF-α controlled the intestinal mucosal inflammation, reduced epithelial lesions and neutrophil infiltration as well as tissue content of TNF-α and oxidative stress. Curiously, infliximab at a dose of 5 mg/kg achieves better histological results and produces higher reduction of the levels of TNF-alpha than at a dose of 10 mg/kg with higher reduction of tissue lipid peroxidation than at a dose of 15 mg/kg^27^. Nonetheless, experimental studies have shown that the use of anti-TNF-α is effective in the treatment of chemically induced colitis and to the best of our knowledge to date the use of anti-TNF-α (infliximab) has never been evaluated in experimental models experimental or patients with the severe forms of DC, which makes this study a pioneer. Author's employing experimental models of CD have shown that there is an increase in TNF-α level in the colonic segments without fecal stream^7^ ^,^ ^17^. Therefore, the use of infliximab in experimental models of DC or in patients presenting the severe forms of the disease seems to be a therapeutic strategy that deserves to be evaluated. If the substance proves to be effective experimentally on DC, it may be used with an effective therapeutic strategy for the treatment of patients with severe forms of DC.

The choice of doses of infliximab that we use in this work is based on a literature report using the TNBS-induced colitis model showed that the dose of 5 mg/kg presented better results when compared to higher doses such as 10 mg/kg and 15 mg/kg^27^. Using this dosage, we observed that all of the inflammatory parameters considered to compose the inflammatory score (epithelial atrophy, epithelial loss, presence of crypt abscesses and inflammatory infiltrate) were not modified in the colonic segments with fecal stream (with a supply of AGCCs preserved). These parameters did not change independent of the intervention solution (saline or infliximab) as well as the infliximab concentration used. When we used these variables to set up the inflammatory score, we verified that there were no significant changes, regardless of the substance or concentration employed. These results demonstrate the importance of the normal supply of SCFAs in the preservation of intestinal mucosal epithelium as well as in the prevention of inflammation. Curiously, we observe decrease in neutrophil infiltrate, evaluated by tissue expression of MPO in colonic segments with preserved fecal stream in animals treated only with infliximab, particularly at doses of 10 mg/kg. It is possible that systemic action of infliximab can reduce de levels of neutrophil infiltrate in colonic epithelium even when fecal transit is maintained.

When we evaluated the histological changes in the colonic segments devoid of fecal stream, the results changed significantly. In animals treated with infliximab, regardless of the concentration used (5 mg/kg/week or 10 mg/kg/week), there was a significant reduction in the inflammatory score when compared to the animals submitted to intervention with saline. The benefits of the use of infliximab therapy are even more evident when we consider the evaluation of the neutrophil infiltrate assessed by the tissue content of MPO. In all animals treated with infliximab, we verified a significant reduction in the tissue levels of MPO, regardless of the concentration used, when compared to those submitted to intervention with saline. Differently from what has been shown by other authors who evaluated the efficacy of infliximab in experimental models of TNBS-induced colitis, in this study we identified greater reduction of MPO levels in infliximab-treated animals especially when higher concentrations were used (10 mg/kg/weeks). These findings may be related to the lower intensity of the inflammatory process in DC models when compared to experimental models of chemically TNBS-induced colitis^24^. The efficacy of infliximab was maintained independent of the concentration employed and the improvement of the neutrophil infiltrate was related to the improvement of the parameters considered in stratification of inflammatory score. These results suggest that therapy with infliximab proved to be effective in regression of colonic mucosa neutrophilic infiltrate devoid of fecal stream. These findings are similar to those found when infliximab was used in experimental models of TNBS induced-colitis suggesting that biological therapy may be used for the treatment of severe clinical forms of human DC.

## Conclusions

The results found in this experimental study show that therapy with infliximab reduces the inflammation and the neutrophil infiltrate in colonic segments devoid of the fecal stream. These results allied to the recognized efficacy of infliximab therapy in the treatment of IBD suggest that the use of the substance in patients with severe forms of DC is a promising therapeutic strategy that deserves to be evaluated.
